# Report of the Diseases of Edinburgh for January, 1809

**Published:** 1809-03

**Authors:** John Roberton


### *Report of the Diseases of Edinburgh for January,* 1809.

By John Robertson, M. D.

This month commenced with a heavy fall of snow and rain, and at the same time with very heavy gales of wind. In this state the weather continued for about five or six days, when a general thaw occurred, by which the greater part of the snow, in the course of two days, was almost completely dissolved. This was succeeded by intense frost, with occasional falls of snow, till about the middle of the month. The weather still continued excessively cold, and within a few days of the end of the month, another very heavy fall of snow, accompanied by a hurricane of wind, occurred. This was, in one night, by a general thaw without rain, nearly dissolved, and the month terminated with two or three days of rather favourable weather.

The barometer, about the middle of the month, during the rain and snow, fell considerably; but towards the end it in general stood high, though it was variable.

Near the last third of the month the thermometer stood for several days, at least 18 degrees below the freezing point, and for some days more was never less than 12 degrees below freezing. During the few days of favourable weather, however, which happened near the end of the month, the thermometer was in general as high as from 40 to 50 degrees.

Stomach and bowel complaints have been more or less prevalent throughout the whole of the month; indeed, from the irregularity of living, exposure to cold and dampness, states of weather not unfrequent in our variable climate about this time, and from the immense quantity of

Whiskey

Whiskey consumed at the same time by certain classes of people, these complaints invariably visit us every year.— Regularity in living, with the administration of emetics and purgatives, almost invariably remove them.

Catarrh and cynanche tonsillaris have been of frequent occurrence; and the first of these, in a chronic state, with chronic rheumatism, asthma, &c. have been very common among certain classes of people. Indeed, with but few exceptions, the most prevalent diseases have been of an inflammatory nature, and, in many instances, uncommonly severe. Various cases of pneumonia and enteritis have, independently of the most active treatment, terminated fatally. In some instances of both these complaints, I have found it necessary, in the same patient, to employ every remedy either recommended by authors or found useful in practice, without experiencing the least benefit, although after a large bleeding, or the application of a very large blister over the affected parts, the patient seemed to experience a remarkable alleviation of the more alarming symptoms; yet in a few hours, they all returned with redoubled severity. Such patients had not only to strive with the severity of these symptoms, but, from the large evacuations which had been found absolutely necessary, the general debility with which they were also affected, independently of the original disease, was of itself sufficient to endanger their lives.

Hydrocephalus, or rather that inflammatory state of the system which precedes the effusion of water into the ventricles of the brain, has been uncommonly prevalent; indeed much more so than I have observed at almost any former period. People in this part of the world are now beginning to observe that this complaint is one, not of rare, but of *very frequent* occurrence; they therefore at once decide that it is a new disease with which they are visited, without reflecting that the frequency of its occurrence is not owing to the absolute frequency of the disease, but to our more perfect knowledge of those symptoms which indicate its presence. That large proportion of individuals who are perpetually grumbling and finding fault with every thing, sagely attribute the unusually frequent appearance of this disease to the introduction of that *diabolical* practice, the inoculation for the cow-pox! and thus endeavour, *but in vain,* not merely to reduce one of the most important modern improvements to a state of insignificance, but positively to transmute it into the cause of the greatest of injuries. The absurdity of such nonsense must at once appear too ridiculous to require an attempt at refutation on on my part. Like the objections which were warmly urged even to the inoculation for the natural small-pox, when it was first proposed, it will have its day, and will, like them, be soon consigned to eternal oblivion. But to return to the subject of hydrocephalus, I may observe, that the practice which I have elsewhere recommended, is not only absolutely necessary during the early stages of that affection, when the system is in the state of greatest vigor, but even when debility has supervened in no inconsiderable degree; when we find, from the pulse, that the action of the vascular system is still greatly accelerated, we are warranted to take blood from the temples or jugular vein, till this state is somewhat changed. About a fortnight ago, I visited a child, where the propriety of the above mentioned practice was clearly exemplified. This child was affected with all the more common inflammatory symptoms incident to the early stages of hydrocephalus internus. The usual application of a cap blister was made, and leeches were applied to the temples, with the administration of brisk purgative medicines in consequence of the bowels being constipated. These, however, produced only temporary relief, for still the pulse continued generally full and much more frequent than natural, yet the bodily strength gradually declined, and the child, in the course of four days, was greatly reduced in strength, so much so, that it was supposed by every one present to be almost in articulo mortis. At this important moment, when scarcely a hope of success was to be expected, I conceived it proper to make the last effort in my power; I instantly applied six leeches to the temples, and ordered that the child should be frequently immersed in the warm bath. In consequence of this, the blood was profusely discharged, and, in about an hour after it, the child, which had scarcely shewn a symptom of animation for several hours before, began to cry, the blistered part being still kept open by epispastic ointment, and, in the course of the following day, the child seemed greatly relieved. In two days more, the pulse was reduced not only in frequency but in strength, and the child has now, without any further treatment, completely recovered, having only that general debility to strive against, which time and attention will in all probability very speedily remove.

*4, St. James's Street.*

